# Quantification of Rat Kisspeptin Using a Novel Radioimmunoassay

**DOI:** 10.1371/journal.pone.0097611

**Published:** 2014-05-20

**Authors:** James S. Kinsey-Jones, Kylie E. Beale, Joy Cuenco, Xiao Feng Li, Stephen R. Bloom, Kevin T. O’Byrne, Kevin G. Murphy

**Affiliations:** 1 Section of Investigative Medicine, Department of Medicine, Imperial College London, London, United Kingdom; 2 Division of Women’s Health, School of Medicine, King’s College London, London, United Kingdom; University of Córdoba, Spain

## Abstract

Kisspeptin is a hypothalamic peptide hormone that plays a pivotal role in pubertal onset and reproductive function. Previous studies have examined hypothalamic kisspeptin mRNA expression, either through *in situ* hybridisation or real-time RT-PCR, as a means quantifying kisspeptin gene expression. However, mRNA expression levels are not always reflected in levels of the translated protein. Kisspeptin-immunoreactivity (IR) has been extensively examined using immunohistochemistry, enabling detection and localisation of kisspeptin perikaya in the arcuate nucleus (ARC) and anteroventral periventricular nucleus (AVPV). However, quantification of kisspeptin-IR remains challenging. We developed a specific rodent radioimmunoassay assay (RIA) capable of detecting and quantifying kisspeptin-IR in rodent tissues. The RIA uses kisspeptin-10 as a standard and radioactive tracer, combined with a commercially available antibody raised to the kisspeptin-10 fragment. Adult female wistar rat brain samples were sectioned at 300 µm and the ARC and AVPV punch micro-dissected. Brain punches were homogenised in extraction buffer and assayed with rodent kisspeptin-RIA. In accord with the pattern of kisspeptin mRNA expression, kisspeptin-IR was detected in both the ARC (47.1±6.2 fmol/punch, mean±SEM n = 15) and AVPV (7.6±1.3 fmol/punch, mean±SEM n = 15). Kisspeptin-IR was also detectable in rat placenta (1.26±0.15 fmol/mg). Reverse phase high pressure liquid chromatography analysis showed that hypothalamic kisspeptin-IR had the same elution profile as a synthetic rodent kisspeptin standard. A specific rodent kisspeptin-RIA will allow accurate quantification of kisspeptin peptide levels within specific tissues in rodent experimental models.

## Introduction

The kisspeptins are a family of peptides essential for the onset of puberty and the regulation of reproductive function. The absence of a functional kisspeptin receptor (KiSS1r) or *Kiss-1* gene results in low gonadotrophin levels and failure to undergo pubertal development in both mice and humans [1]–[4]. Administration of exogenous kisspeptin potently stimulates gonadotropin secretion in rodents and man [4]–[7]. Kisspeptin is the endogenous ligand of the G-protein coupled receptor Kiss1r [8]–[10]. The stimulatory effects of kisspeptin on the reproductive axis appear to be mediated by a direct activation of KiSS1r-expressing gonadotrophin releasing hormone (GnRH) neurons [11]–[13].

In humans, *KISS1* encodes a 145 amino acid precursor peptide which is cleaved to form a 54-amino-acid peptide, known as kisspeptin-54 or metastin [10], and shorter fragments 14, 13 and 10 amino acids long [9]. All kisspeptin share a common 10 amino acid C-terminal section, which is required for kisspeptin receptor activation. Kisspeptin-54, -14, -13 and -10 all bind to Kiss1 with equal affinity and efficacy *in vitro* [9]. The largest proteolytic product in rodents is a 52 amino-acid peptide. Although this peptide shares only relatively low overall homology (52%) with human kisspeptin-54, the C-terminal 10 amino-acid signalling sequences is highly conserved between mouse and human *KiSS1* proteins, varying by only one amino acid [Tyr 10 (Y) in rodents to Phe 10 (F) in humans] [14].

Expression of *Kiss1* mRNA has been observed in tissues including the placenta, pancreas, small intestine, and brain in humans [8]–[10]. Within the human CNS, kisspeptin immunoreactive cell bodies are predominantly located in the infundibular nucleus of the hypothalamus [15]. In rodents, kisspeptin perikarya are located in two major populations in the hypothalamus, the anteroventral periventricular nucleus (AVPV) and the arcuate nucleus (ARC) [5], [16].

Kisspeptin mRNA expression has been quantified using *in situ* hybridisation (ISH) and quantitative polymerase chain reaction (qPCR). This provides valuable information on gene transcription, but it is widely recognised that changes in mRNA are not always mirrored by protein levels [17], [18]. In humans, circulating kisspeptin levels have been quantified by radioimmunoassay, and immunocytochemical (ICC) methods have been used to localise kisspeptin expression in humans and rodents [7], [19]. However, to date, kisspeptin peptide levels in the rodent have not been measured. Western blotting can be used to measure protein levels, but it can be challenging to detect small changes in kisspeptin–IR levels using this technique. In the current study we have developed a sensitive and specific radioimmunoassay (RIA) which enables the quantification of kisspeptin levels within rodent tissues.

## Materials and Methods

### Materials

Synthetic kisspeptin-10 was obtained from the Advanced Biotechnology Centre, Imperial College, London, UK. Kisspeptin-52 was purchased from Phoenix Pharmaceuticals (Burlingame, CA, USA). The kisspeptin polyclonal antibody was purchased from Millipore (Watford, UK).

### Rat Tissue

Female Wistar rats (220–250 g) obtained from Charles River (Margate, UK) were housed in single cages (specific pathogen free, Imperial College London, UK) and maintained in a controlled environment (temperature 21–23°C, 12-h light–dark cycle, lights on at 07∶00) with *ad libitum* access to food (RM1 diet; SDS UK, Witham, Essex, UK) and water. All animal procedures were approved by the British Home Office Animals (Scientific Procedures) Act 1986. Estrus cyclicty was monitored daily by vaginal lavage, slides were examined under a light microscope to determine the dominant cell type. On the morning of the diestrus phase of the cycle, animals were killed by decapitation, and either whole brain or hypothalamus dissected and snap frozen then stored at −80°C. Pregnant female rats were obtained from Charles River. At pregnancy day 20 rats were killed and the placenta removed, snap frozen and stored at −20°C. An additional cohort of female Wistar rats were bilaterally ovarectomized (OVX) and implanted with an estradiol (E_2_) filled (150 ug E_2_/ml) silicone capsule. This model has previously been shown to mediate changes in kisspeptin expression [20]. Two weeks later animals were killed by decapitation and whole brain dissected, snap frozen and stored at −80°C.

### Radioimmunoassay

An RIA directed against rodent kisspeptin was developed. The rabbit kisspeptin-10 polyclonal antibody used has been demonstrated to detect markedly diminished immunoreactivity in *Kiss1* ^−/−^ mice compared to wild type mice when used for immunocytochemistry [21], and to have minimal cross-reactivity with other RF-amide peptides [22]. ^125^I-kisspeptin-10 label was prepared using the chloramine-T method and purified by reverse-phase high-performance liquid chromatography (RP-HPLC) on a C18 column (Waters, Milford, MA) over a 32–38% 90-min gradient of acetonitrile (AcN)/water/0.1% trifluoroacetic acid (TFA). The specific activity of kisspeptin label was 56 Bq/fmol. The assay was set up as previously described [23]. The final antibody dilution used was 1∶87 000. Kisspeptin-10 label was used at 20 Bq/tube. The assay was performed in 0·06 M phosphate buffer (pH 7·2) containing 0·3% (v/v) bovine serum albumin (BSA) and 0·02% (v/v) Tween 20. After 3 days of incubation at 4°C, antibody-bound and free fractions were separated by charcoal (2.4 g charcoal, 0.24 dextran in 100 ml phosphate buffer) adsorption of the free fraction. Unless stated, all tissues were homogenised using a Kimble Kontes pellet pestle (Sigma-Aldrich, Dorset, UK) in 450 µl of acid ethanol extraction buffer (0.15% hydrochloric acid in 25% ethanol) [24]. To assess parallelism of the assay, standard curves of 1, 2, 3, 5, 10, 15, 20, 30, 50 and 100 fmol/tube of kisspeptin-10 and kisspeptin-52 were set up in duplicate in conjunction with dilution curves of hypothalamus tissue extracts (5, 10, 20 and 30 µl of extract, also in duplicate) (n = 5). To assess the recovery efficiency of the assay concentrations from 50 to 200 fmol/tube kisspeptin-10 were added to 450 µl rat cerebellum tissue extract and assayed. For the quantification of kisspeptin-IR in tissues, a standard curve consisting of 1, 2, 3, 5, 10, 15, 20, 30, 50 and 100 fmol/tube kisspeptin-10 in duplicate was used.

### Chromatography

Synthetic kisspeptin-52 (2 nmol in 150 ul water) was fractionated using RP-HPLC Phenomenex Jupiter 4 µm Proteo 90 Å column and eluted with a 30–35% gradient of ACN plus 0·05% (v/v) water/0·1% (v/v) 50 min at a flow rate of 1 ml/min per fraction. Fractions were collected every min. An additional set of hypothalamic tissue samples (*n* = 4) was extracted using the protocol described above, centrifuged at 15 000 g for 3 min, and the supernatants filtered through 0·2 µm hydrophilic membranes (Sartorius, Göttingen, Germany). Samples of 150 ul from each extract were then loaded separately onto the HPLC column and eluted under the same conditions described above. Fractions from all runs were freeze-dried, reconstituted in assay buffer and the kisspeptin content determined by RIA.

### Quantification of AVPV, ARC and Placenta Kisspeptin

Several groups have confirmed kisspeptin expression in the placenta and the hypothalamic AVPV and ARC [25], [26]. Brain sections (300** µ**m) were cut on a cryostat, and bilateral punches (1****mm diameter) of the AVPV were taken from Bregma +0.2 to −0.4****mm, single midline punch (1****mm diameter) that included both ARC was taken from bregma −1.7 to −3.9 according to the rat brain atlas [27], following the micropunch method of Palkovits [28]. Hypothalamic punches, cerebellum and placental tissue (350 mg) were homogenised and extracted in acid ethanol buffer as described above, and kisspeptin content measured by radioimmunoassay. A separate set of tissues were also extracted by boiling for 15****min in 0·5 M acetic acid [24].

### Statistical Analysis

All data are presented as mean ± standard error of the mean (SEM). Levels of kisspeptin expression were analysed using an unpaired t-test (GraphPad Prism version 5 for Windows; GraphPad Software, San Diego, CA).

## Results

### Radioimmunoassay

**Figure 1:**
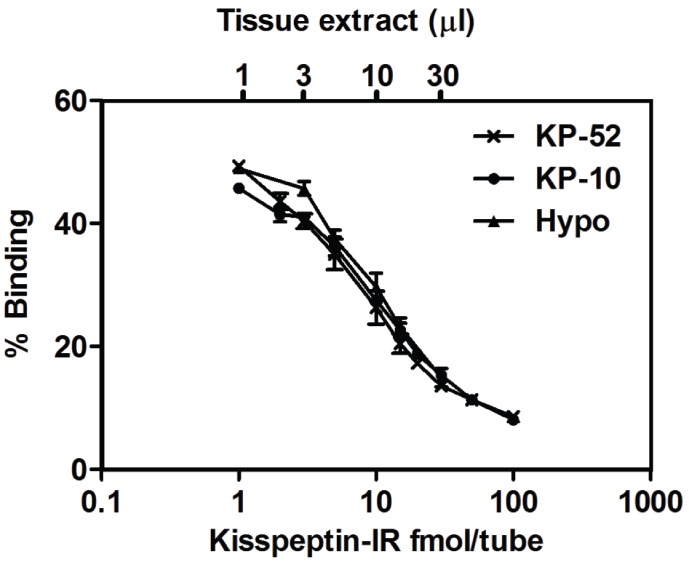
HPLC profile of kisspeptin-immunoreactivity from hypothalamic extracts. Solid lines- Kisspeptin concentration; broken lines-% acetonitrile (ACN). ↓KP-52 represents the elution position of Kisspeptin-52 standard. The recovery of Kisspeptin-IR in the tissue extract from each column run was above 80% (means S.E.M.; n = 4).

The assay demonstrated 100% cross-reactivity to both rodent kisspeptin-10 and rodent kisspeptin-52. The assay had a sensitivity of 0.81±0·12 fmol/tube (mean ± SEM), *n* = 3 with 95% confidence. The least detectable tissue concentration was <1.21 fmol/tube, and the midrange was 18.14 fmol/tube. Inter- and intra-assay variation were established to be 8·2%±0·7 and 6·8%±1·7 respectively, *n* = 5. Recovery of kisspeptin from tissue homogenate was between 78% and 96%. The dilution curve for hypothalamic extract was almost parallel to that of kisspeptin-10 and kisspeptin-52 standard, *n* = 5 (Figure 1).

### Chromatography

**Figure 2:**
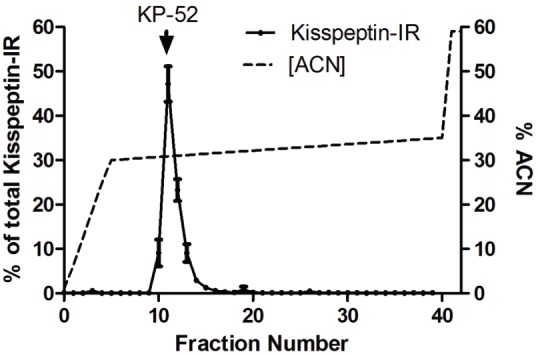
Parallelism of standard and tissue extract for novel rodent Kisspeptin RIA. Kisspeptin **−**10 (KP-10) and hypothalamus (Hypo) (*n* = 5).

HPLC of hypothalamic extracts resulted in a distinct kisspeptin-IR peak at the same point at which synthetic kisspeptin-52 peptide eluted (Figure 2). The recovery of kisspeptin-IR for each column run of the tissue extracts was >80%.

### Quantification of AVPV and ARC Punches and Placenta

**Figure 3:**
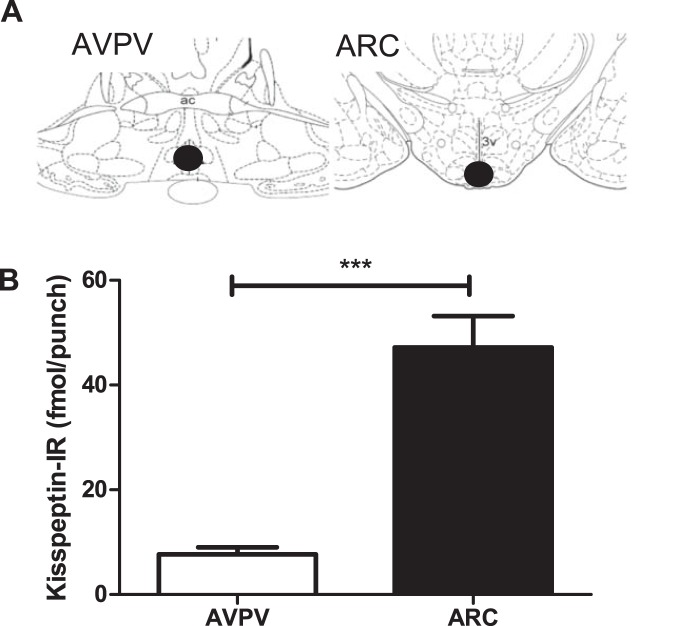
Quantification of kisspeptin-IR in the rat brain (A) Diagram of a rat brain coronal section showing the position of the punch microdissections for the Hypothalamic anteroventral periventricular nucleus (AVPV) and the arcuate nucleus (ARC). AVPV at bregma −0.26****mm and the ARC at bregma −3.30****mm according to the rat brain atlas of Paxinos and Watson. Black circles denote punch positions. Ac, anterior commissure; 3v, third cerebral ventricle. (B) Quantification of kisspeptin-IR in the AVPV and ARC of female rats by RIA. Significantly more kisspeptin-IR was detected in the ARC than in the AVPV. (Mean S.E.M, n = 15**–**16, ***P<0.001, Student’s t-test).

**Figure 4:**
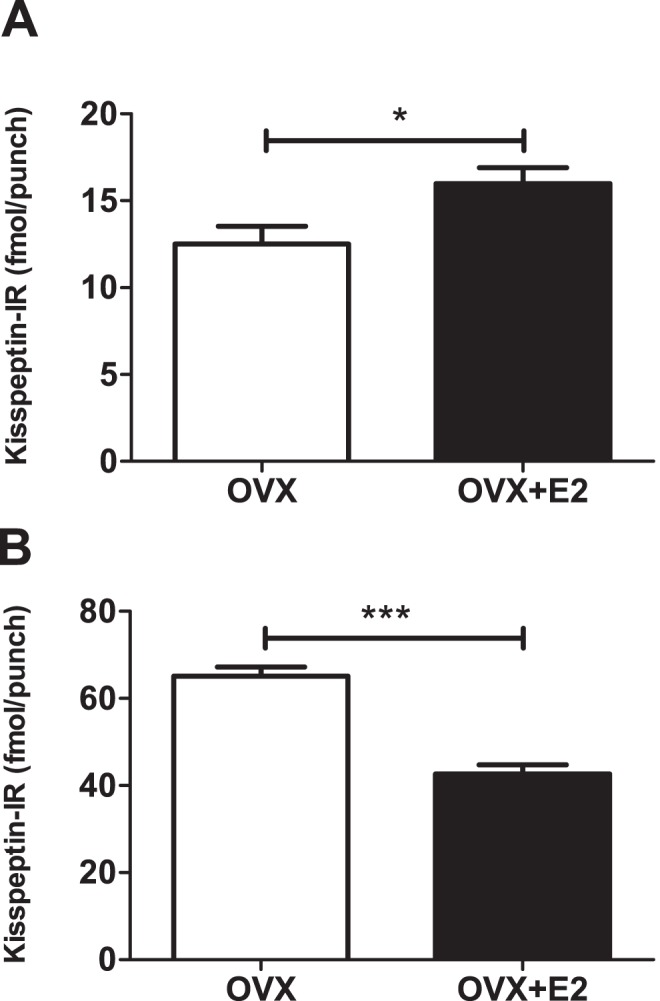
The effect of E_2_ on kisspeptin-IR in the hypothalamus of OVX female rats. (A) E_2_ significantly increased kisspeptin-IR in the AVPV. (B) E_2_ significantly decreased kisspeptin-IR in the ARC. Quantification of kisspeptin-IR by RIA. (Mean S.E.M, n = 6**–**8, *P<0.05, ***P<0.001, Student’s t-test).

Kisspeptin-IR in the acid ethanol extracted AVPV and ARC of female rat was determined. Significantly more kisspeptin-IR was detected in the ARC than in the AVPV (47.1±6.0 vs. 7.6±1.3 fmol/punch, P<0.001, n = 15, Figure 3). As anticipated, a significant increase in AVPV kisspeptin-IR was observed in OVX+E_2_ compared with the OVX rats (16±0.9 vs. 12.5±1 fmol/punch, mean ± SEM P<0.05, n = 6**–**8, Figure 4A), and a significant decrease observed in ARC kisspeptin-IR in OVX+E_2_ compared with OVX rats (43.3±2.9 vs. 66.2±2.1 fmol/punch, mean ± SEM, P<0.001, n = 6**–**8, Figure 4B). We detected 1.26±0.15 fmol/mg (n = 5) kisspeptin-IR in acid ethanol extracted placental tissue. No kisspeptin-IR was detected in the cerebellum tissue. No measurable kisspeptin-IR levels were detected in tissues extracted by boiling for 15****min in 0·5 M acetic acid (data not shown).

## Discussion

In the present study, we have characterised the development of a novel kisspeptin RIA and demonstrated its utility in quantifying kisspeptin-IR within the AVPV and ARC of female rats. We have demonstrated that this assay can detect changes in kisspeptin expression in response to E**_2_** feedback. In accord with studies using other methods, we observed suppression of kisspeptin-IR in the ARC and increased kisspeptin-IR in the AVPV in response to E**_2_** in ovariectomised female rats [29]. Numerous studies have demonstrated a wide distribution of kisspeptin expression throughout the rodent brain [30], [31]. Initial studies examining the distribution of kisspeptin-IR rodents were hampered by the limited specificity of available antibodies [30]. However, the development of reliable antibodies which recognise rodent, sheep [19] and human [7] kisspeptin has enabled the distribution of kisspeptin-IR to be fully mapped. Furthermore, we observe a single IR peak following HPLC of hypothalamic extracts, at the point we would expect kisspeptin-52 to elute, thus supporting previous findings regarding antibody specificity [19].

In addition to mapping kisspeptin distribution, ICC methodologies have been utilised to quantify changes in expression under different experimental and physiological conditions [31], [32]. Kisspeptin-IR has been successfully quantified in the AVPV, and also in the ARC, though the ARC has presented difficulties due to the dense plexus of kisspeptin fibres present [31], [33], [34]. The limitations of ICC preclude the quantification of the absolute number of kisspeptin molecules or its concentration in a sample. Further, though semi-quantitative methodologies provide information about relative quantities of kisspeptin-IR within each study, it is difficult to compare the levels observed between different studies and models. Kisspeptin expression can be quantified using ISH [5] or qPCR [35]. However, it is becoming increasingly evident that examination of mRNA levels may be an unreliable proxy for protein concentrations [36], [37], with only ∼40% protein levels being explained by mRNA abundances in mammalian cell lines [17], [18]. Differences in the distribution of kisspeptin mRNA and immunoreactivity have previously been reported. For example, kisspeptin-IR cell bodies and fibres were described in the dorsomedial nucleus of mice [31], though ISH failed to identify kisspeptin mRNA within this region [26]. A subsequent study suggested that the IR detected within the dorsomedial nucleus may be the result of cross-reactivity of the antibody used with neuropeptide FF [38], though it is interesting to note that a recent study detected kisspeptin-immunoreactivity in some DMN cells in the mouse using a different antibody (Franceschini et al 2013). In the current investigation, the higher level of kisspeptin-IR in the ARC than the AVPV is in accord with the relative levels predicted by assessment of mRNA levels. Kisspeptin-IR was also present in the placenta, albeit at relatively low levels. Previous studies have suggested that the expression of kisspeptin mRNA in the placenta varies during pregnancy, and it may be that higher levels would have been detected at another time point [25]. Our data also suggest that the majority of hypothalamic kisspeptin-IR is in the form of kisspeptin-52, though it is possible that our extraction methods favour this particular form, and that other kisspeptin forms are present at lower levels. Further studies are thus necessary to conclusively demonstrate the relative quantities of the different kisspeptins in the hypothalamus.

In summary, we have developed a novel radioimmunoassay for the measurement of kisspeptin-IR within rat tissue. When combined with the Palkovits punch micro-dissection methodology, this assay enables quantification of kisspeptin-IR within specific brain nuclei.
