# Correction: Quantification of Rat Kisspeptin Using a Novel Radioimmunoassay

**DOI:** 10.1371/journal.pone.0110312

**Published:** 2014-09-30

**Authors:** The PLOS ONE Staff

The figure legends for Figures 1 and 2 are incorrectly switched. The figure legend that appears for Figure 1 should be for Figure 2, and the figure legend that appears for Figure 2 should be for Figures 1. The images appear in the correct order. Please refer to the correct figure legends here

**Figure 1:**
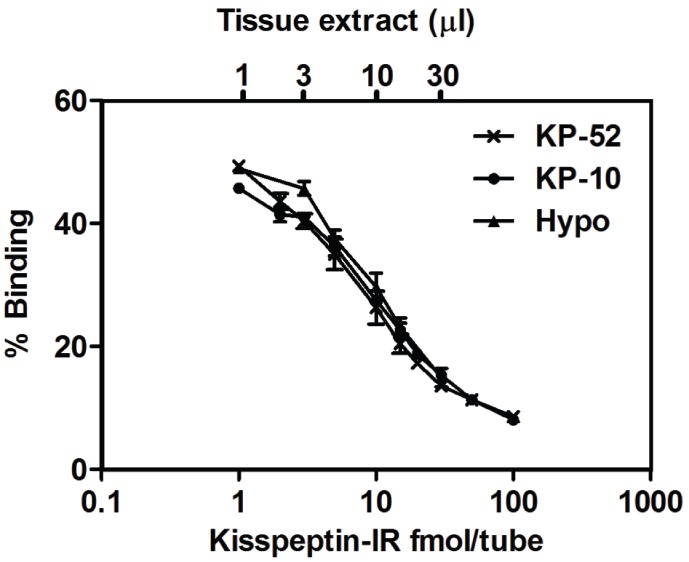
Parallelism of standard and tissue extract for novel rodent Kisspeptin RIA. Kisspeptin −10 (KP-10) and hypothalamus (Hypo) (*n*  =  5).

**Figure 2:**
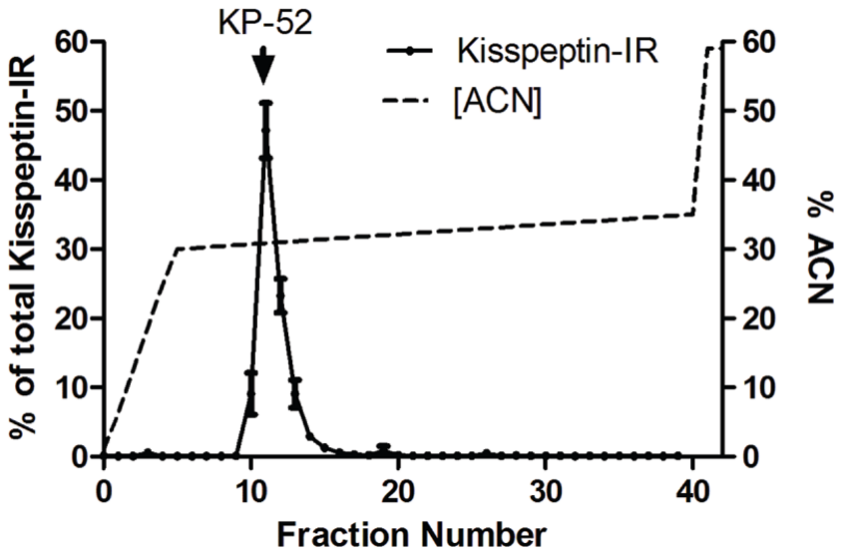
HPLC profile of kisspeptin-immunoreactivity from hypothalamic extracts. Solid lines- Kisspeptin concentration; broken lines-% acetonitrile (ACN). ↓KP-52 represents the elution position of Kisspeptin-52 standard. The recovery of Kisspeptin-IR in the tissue extract from each column run was above 80% (means S.E.M.; n  =  4).

## References

[1] Kinsey-Jones JS, Beale KE, Cuenco J, Li XF, Bloom SR, et al (2014) Quantification of Rat Kisspeptin Using a Novel Radioimmunoassay. PLoS ONE 9(5): e97611 doi:10.1371/journal.pone.0097611 24845101 10.1371/journal.pone.0097611 PMC4028310

